# Investigating the epithelial barrier and immune signatures in the pathogenesis of equine insect bite hypersensitivity

**DOI:** 10.1371/journal.pone.0232189

**Published:** 2020-04-28

**Authors:** Iva Cvitas, Simone Oberhänsli, Tosso Leeb, Martina Dettwiler, Eliane Müller, Remy Bruggman, Eliane Isabelle Marti

**Affiliations:** 1 Division of Experimental Clinical Research, Department of Clinical Research and Veterinary Public Health, Vetsuisse Faculty, University of Bern, Bern, Switzerland; 2 Graduate School for Cellular and Biomedical Sciences, University of Bern, Bern, Switzerland; 3 Dermfocus, Vetsuisse Faculty, University of Bern, Bern, Switzerland; 4 Interfaculty Bioinformatics Unit and SIB Swiss Institute of Bioinformatics, University of Bern, Bern, Switzerland; 5 Institute of Genetics, Department of Clinical Research and Veterinary Public Health, Vetsuisse Faculty, University of Bern, Bern, Switzerland; 6 Institute of Animal Pathology, Department of Infectious Diseases and Pathobiology, Vetsuisse Faculty, University of Bern, Bern, Switzerland; 7 Department of Biomedical Research, Molecular Dermatology and Stem Cell Research, University of Bern, Bern, Switzerland; 8 Department of Dermatology, Inselspital, Bern University Hospital, Bern, Switzerland; INSERM, FRANCE

## Abstract

Insect bite hypersensitivity (IBH) is a Th-2, IgE-mediated dermatitis of horses caused by bites of insects of the genus *Culicoides* that has common features with human atopic dermatitis. Together with Th-2 cells, the epithelial barrier plays an important role in development of type I hypersensitivities. In order to elucidate the role of the epithelial barrier and of the skin immune response in IBH we studied the transcriptome of lesional whole skin of IBH-horses (IBH-LE; n = 9) in comparison to non-lesional skin (IBH-NL; n = 8) as well as to skin of healthy control horses (H; n = 9). To study the "baseline state" of the epithelial barrier, we investigated the transcriptome of non-lesional epidermis in IBH-horses (EPI-IBH-NL; n = 10) in comparison with healthy epidermis from controls (EPI-H; n = 9). IBH-LE skin displayed substantial transcriptomic difference compared to H. IBH-LE was characterized by a downregulation of genes involved in tight junction formation, alterations in keratins and substantial immune signature of both Th-1 and Th-2 types with particular upregulation of *IL13*, as well as involvement of the hypoxic pathway. IBH-NL shared a number of differentially expressed genes (DEGs) with IBH-LE, but was overall more similar to H skin. In the epidermis, genes involved in metabolism of epidermal lipids, pruritus development, as well as *IL25*, were significantly differentially expressed between EPI-IBH-NL and EPI-H. Taken together, our data suggests an impairment of the epithelial barrier in IBH-affected horses that may act as a predisposing factor for IBH development. Moreover, these new mechanisms could potentially be used as future therapeutic targets. Importantly, many transcriptional features of equine IBH skin are shared with human atopic dermatitis, confirming equine IBH as a natural model of skin allergy.

## Introduction

Insect bite hypersensitivity (IBH) is the most common allergic skin disease affecting horses. IBH is a seasonally recurrent, highly pruritic dermatitis caused by IgE-mediated hypersensitivity to bites of insects of the genus *Culicoides* [1–3]. Although the allergens differ, IBH has overlapping features with human atopic dermatitis.

Affected horses develop distinguishable lesions typically distributed along the dorsal midline, particularly at the basis of the mane and tail, and less commonly on the ventral midline, head and legs. Initially, lesions are seen as papules, edema and tuft hair which, due to severe pruritus, rapidly progress to crusts, dandruff, alopecia, excoriations, and lichenification. Moreover, lesions can be further exacerbated by secondary bacterial infections [4]. Histologically, IBH is characterized by mixed perivascular to diffuse cellular infiltrates of mononuclear cells and eosinophils in the dermis. Increased numbers of mast cells as well as MHC class II positive cells are found. Additional features are marked acanthosis and hyperkeratosis of the epidermis [1, 5].

Several studies have described IBH as a type I, IgE dependent hypersensitivity. The main mediators of type I hypersensitivities are T helper type 2 cells (Th-2) and their signature cytokines IL-4, IL-5 and IL-13.These cytokines induce isotype (class) switching of B cells and subsequent production of allergen specific IgE antibodies which bind to mast cells, as well as activation and infiltration of eosinophils. Activated mast cells and eosinophils are responsible for development of clinical signs of early-phase reaction. An imbalance between the Th-2 and regulatory T cell (Treg) response in IBH was demonstrated in allergen-stimulated PBMC as well as locally in the skin [6, 7].

Together with Th-2 cell involvement, recent data suggests that epithelial barrier defects play an important role in the development of type I hypersensitivities in humans. In humans, the discovery that loss of function variants in the *FLG* gene encoding filaggrin predispose individuals to develop AD has, for the first time, demonstrated the role of an altered epithelial barrier in the pathogenesis of allergies [8]. Genetic variants in filaggrin affect the terminal differentiation of keratinocytes and therefore impair the epithelial barrier, making it more permeable for different allergens [9, 10]. Moreover, epithelial cells such as keratinocytes are far beyond a mere barrier building cell type. In fact, they produce cytokines and chemokines that possibly play a crucial role in the development of allergic inflammation. Epithelial cells can produce cytokines such as thymic stromal lymphopoietin (TSLP), IL-25, and IL-33. These cytokines have the ability to influence dendritic cells (DCs) that in turn affect proliferation and differentiation of naïve T cells into Th-2 subtype producing IL-4, IL-5, IL-13 and TNF-α [11–15]. Additionally, these cytokines cause *in vivo* expansion of innate lymphoid cells type 2 (ILC2s) that are an important early source of the type-2-cell-associated cytokines IL-5 and IL-13, and to a lesser extent, IL-4. The ILC2s themselves are capable of controlling a type 2 innate immune response, but often collaborate with CD4+ Th-2 cells in order to develop fully blown type 2 immunity [14, 16–18].

Although IBH is the most frequent type I allergic skin disease of horses, the role of the epithelial barrier and skin immune response in the pathogenesis of this disease has not been thoroughly studied. Moreover, a lot of our current knowledge on the pathogenesis of type I hypersensitivities is based on mouse models of the disease. Therefore, studying equine IBH, which is a spontaneously-occurring, natural model of allergy, can contribute to the understanding of human allergies.

We first aimed to understand the role of epithelial barrier in the pathogenesis of IBH, as well as to characterize the type of immune response involved. Therefore, we studied differences in gene expression by comparing the transcriptome of lesional whole skin samples of IBH-affected horses (IBH-LE) in comparison to non-lesional skin (IBH-NL) as well as to skin of healthy control horses (H). Furthermore, we aimed to investigate possible epithelial barrier defects that may be predisposing factors for IBH development, thus we studied the transcriptome of non-lesional epidermis (EPI-IBH-NL) of IBH-affected horses in comparison with epidermis from healthy control horses (EPI-H).

## Materials and methods

### Sample collection

This study was approved by the Animal Experimental Committee of the Canton of Berne, Switzerland (No. BE 69/18). Horses suffering from IBH were diagnosed based on recurrent clinical signs of IBH. The diagnosis was confirmed by histopathological analysis of skin samples. Samples were collected from 8 horses slaughtered due to IBH and two clinical patients suffering from IBH. In these patients, two 8 mm punch biopsies were taken from lesional and non-lesional skin after sedation with detomidine hydrochloride (0.01 mg/kg iv; Domosedan, www.vetoquinol.ch) and local subcutaneous injection of lidocaine. Samples from 9 horses without clinical signs or history of IBH, slaughtered due to reasons not related to skin diseases were collected. An overview of the samples taken is given in S1 Table.

#### Whole skin

Samples from IBH lesional skin (IBH-LE, n = 9) were collected from the dorsal midline, non-lesional skin (IBH-NL, n = 8) from the inner thigh. Samples from control horses were collected from the inner thigh (H, n = 9). After collection, one part of the sample was immediately cut in half, submerged in RNAlater (ThermoFischer Scientific, Waltham, Massachusetts, USA), and stored at 4°C for 24 h. Subsequently, samples were removed from RNAlater and stored at -80°C until used. To confirm the clinical diagnosis, another part of the sample was fixed in 10% formalin (ThermoFischer Scientific) for 24 h, trimmed, dehydrated and embedded into paraffin wax. 2 μm sections of all formalin-fixed and paraffin-embedded skin samples were mounted on glass slides and stained with hematoxylin and eosin (HE) according to routine procedures. The HE slides were evaluated by one board-certified veterinary pathologist without the knowledge of disease history and anatomic location.

#### Epidermis

For RNA sequencing of epidermis, another part of the skin samples (inner thigh) of non-lesional skin (n = 10) from IBH-affected horses and from control horses (n = 9) was processed aseptically after sampling. These samples were incubated at 4⁰C for 24 h with 10 mg/ml Dispase II (Roche, Basel, Switzerland) in Williams E medium (Bioconcept, Allschwil, Switzerland). Subsequently, the epidermis was separated from the dermis using sterile forceps as described [19, 20]. 30 mg of epidermal tissue was frozen in RLT lysis buffer (RNeasy Mini Kit; Qiagen, Hilden, Germany). Samples were kept at -80⁰C until further processed.

### Isolation of RNA and cDNA synthesis

Total RNA was isolated from IBH-LE, IBH-NL and H control whole skin using RNeasy Fibrous Tissue Kit (Qiagen,). Prior to RNA extraction, skin samples were homogenized in 600 μL of RLT lysis buffer (Qiagen) using MagNa tissue lyser (Roche). Samples were homogenized for 45 s with ceramic beads (Roche, Basel, Switzerland) at the shaking speed of 6,500/min, followed by 2 min cooling on ice. Homogenization was repeated for another 30 s at shaking speed of 6,500/min and subsequent 2 min cooling on ice. Supernatants were loaded onto a spin column (QIAshredder, Qiagen) and centrifuged at 16,000x g for 2 min (Qiagen).

Total RNA was isolated from epidermis using RNeasy Mini Kit (Qiagen, Hilden Germany) according to the manufacturer's instructions. Prior to RNA extraction with RNeasy Mini Kit, cell lysates were loaded onto a spin column (QIAshredder, Qiagen) and centrifuged at 16000x g for 2 min.

Contaminating genomic DNA was removed by on-column DNase treatment in samples from epidermis and whole skin. Total RNA was quantified spectrophotometrically at 260 nm (NanoDrop 2000c; ThermoScientific, Reinach, Switzerland) and RNA samples were stored at -80⁰C until used. RNA quality was determined using Fragment Bioanalyzer (Labgene, Châtel-Saint-Denis, Switzerland).

### RNA sequencing

Illumina TruSeq stranded mRNA libraries were prepared according to the manufacturer’s protocol (Illumina, San Diego, USA). Between 14 and 34 million 2 x 50 bp read-pairs per sample were collected on an Illumina NovaSeq 6000 instrument. The quality of the RNA-seq data was assessed using fastqc v. 0.11.5 and RSeQC v. 2.6.4.

### Mapping to reference genome and differential gene expression analysis

The reads were mapped to the reference genome (EquCab3.0) using HiSat2 v. 2.1.0. FeatureCounts v. 1.6.0 was used to count the number of reads overlapping with each gene as specified in the genome annotation (NCBI Equus caballus Annotation Release 103). The Bioconductor package DESeq2 v. 1.18.1 was used to test for differential gene expression between the experimental groups. The Benjamini Hochberg method was used for multiple test correction. We did not remove any genes with low or no expression before running the DESeq analysis as the tool’s “result” function performs an “independent filtering” by default which is based on the mean of normalized counts (see DESeq2 documentation on Bioconductor). The datasets generated during the current study will be available in the ENA repository via accession numbers (xxx). Genes with a false discovery rate (= padjusted) smaller than 0.05 where considered significantly differentially expressed.

### Gene ontology analysis

TopGo v. 2.24.0 was used to identify gene ontology terms significantly enriched for differentially expressed genes (threshold for genes to be significantly differentially expressed: padjusted < 0.05). All tests were repeated using different combinations of algorithm (weight01 or classic) and test statistic (Fisher or Kolmogorov-Smirnov) to assess the robustness of the results. An interactive Shiny application was set up to facilitate the exploration and visualisation of the RNA-seq analysis results. All analyses were run in R version 3.4.4 (2018-03-15).

### Pathway analysis

To visualize differences in gene expression between conditions within biochemical pathways entrezgene ids of significantly differentially expressed genes from each comparison (epidermis: padjusted < 0.05, IBH-LE vs H and IBH-LE vs IBH-NL: padjusted < 0.05) were mapped to KEGG pathways of horse (species =“ecb”, analysis date = July 2019) using the R Bioconductor packages “KEGGREST” and “pathview” (R version 3.5.0, pathview v. 1.22.3, KEGGREST v 1.22.0). Expression changes (log 2 fold change) were visualized with colors blue (negative log fold change) and red (positive log fold change). Additionally, Reactome analysis (https://www.reactome.org) was performed based on gene symbols of significantly differentially expressed genes with p values as mentioned above, for comparisons of IBH-LE vs H whole skin and and non-lesional IBH epidermis vs healthy epidermis [21].

ClusterProfiler v3.10.1 was used to test for enrichment of KEGG pathways with significantly differentially expressed genes. Gene set enrichment analysis (GSEA) was performed using the gseKEGG-function (default settings except for minGSSize = 50) and a ranked list as input (entrezgene-id and it’s corresponding–log 10 (raw pvalue) x +/-1 (depending on the direction of the foldchange), list sorted according to–log10 (raw pvalue) x +/-1 (depending on the direction of the foldchange).

## Results

The histopathological evaluation revealed inflammatory changes compatible with a type 1 hypersensitivity in all IBH-LE samples, thus confirming the clinical diagnosis of IBH. H skin samples were devoid from inflammation. IBH-LE skin samples were characterized by substantial hyperkeratosis and acanthosis, infiltration with lymphocytes and a strong infiltration with eosinophils in all but one sample. This sample was characterized by a strong dermal infiltration with lymphocytes. Substantial hyperkeratosis and acanthosis were not observed in IBH-NL skin, however, IBH-NL samples also showed infiltration with eosinophils, although to a lower degree than IBH-LE skin.

### Transcriptome analysis of whole skin

Sequencing data could be generated from all samples. Out of 33,078 genes annotated in the reference genome, 21,337 (IBH-LE vs H skin), 20,328 (IBH-LE vs IBH-NL skin) and 21,337 (IBH-NL vs H skin) genes showed sufficient expression (i.e. read counts) to be included in the analysis of differential expression in the whole skin.

**Fig 1:**
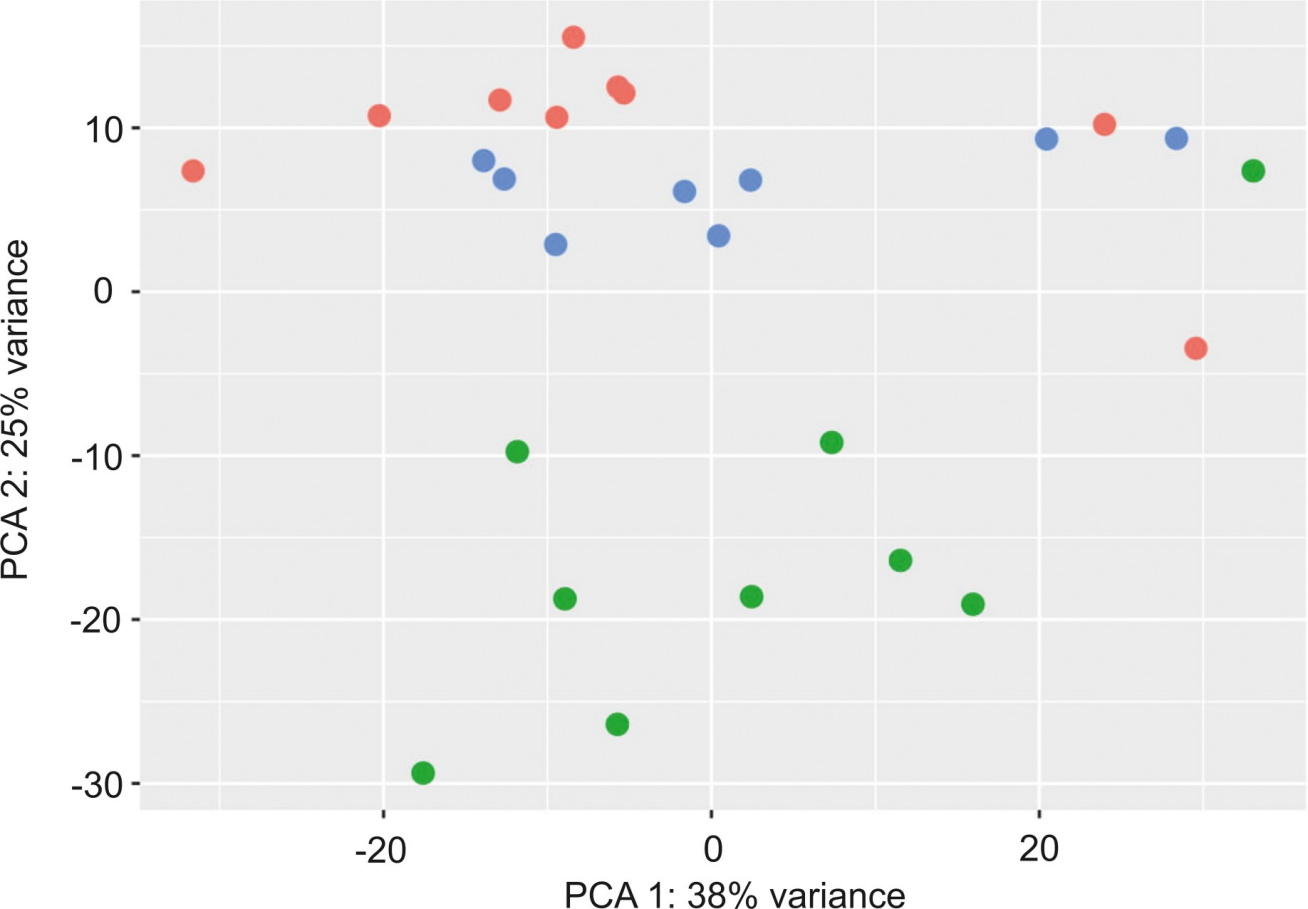
Principal component analysis of top 500 most variable DEGs in the first two component spaces (p<0.05): Lesional whole skin of IBH-affected horses (IBH-LE) is shown in green, non-lesional whole skin of IBH-affected horses (IBH-NL) in blue and healthy skin of control horses (H) in red.

Principal component analysis (PCA) of IBH-LE, IBH-NL and H whole skin, based on expression data of 500 genes with the highest variability, showed a clear separation between IBH-LE and H skin samples (Fig 1). Samples of IBH-NL clustered between the IBH-LE and H samples, in closer proximity to the H samples (Fig 1).

**Fig 2:**
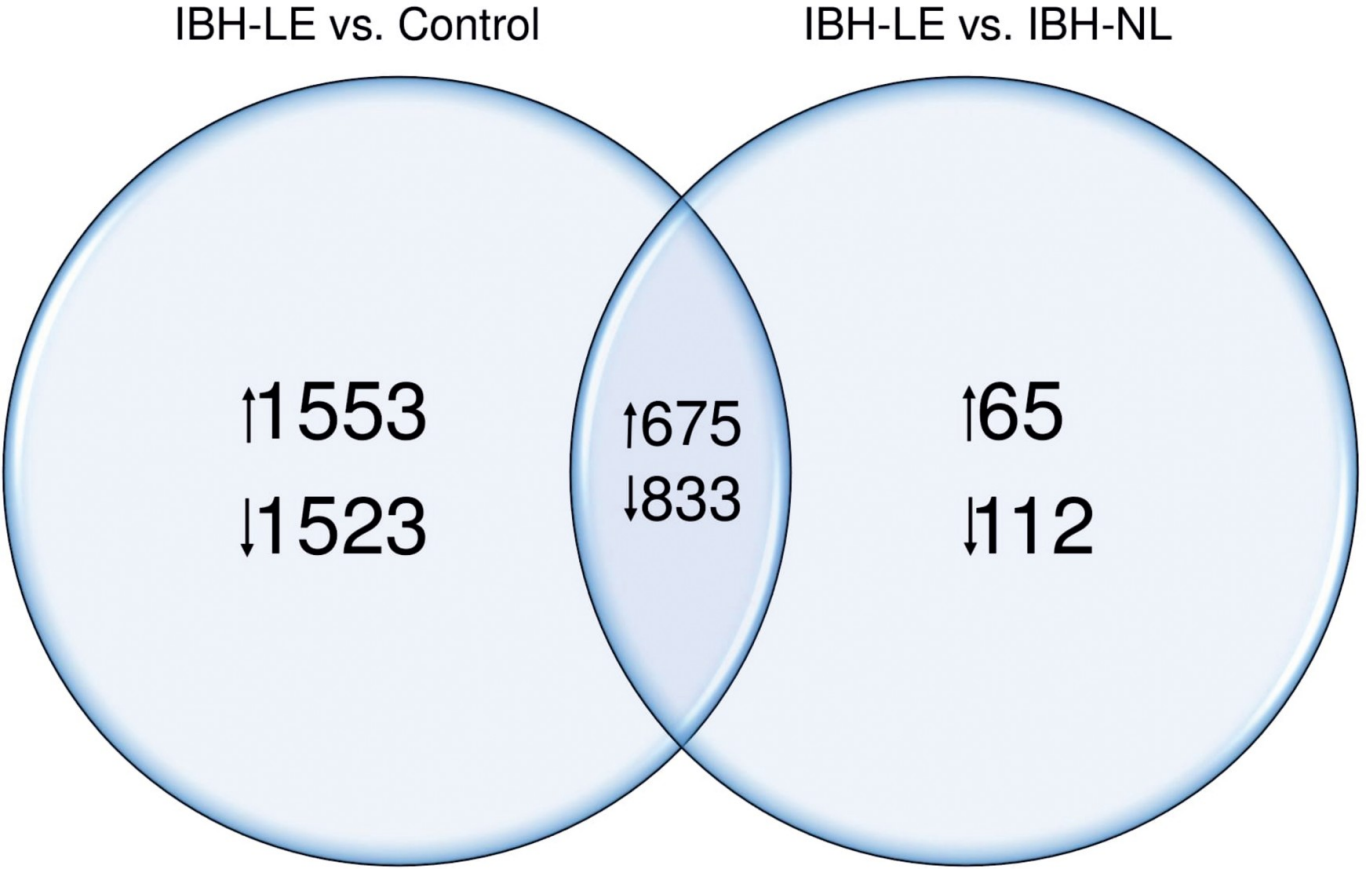
Venn diagram of DEGs shared between IBH-LE vs. H and IBH-LE vs. IBH-NL comparisons (p<0.05). The numbers of up- and down regulated genes are indicated.

Comparing IBH-LE to H skin, we found 2,228 significantly upregulated (range log2 fold change 0.14–2.9) and 2,356 significantly downregulated genes (range log2 fold change -0.17 - -2.74) (S1A Fig and S2 Table). When we compared IBH-LE to IBH-NL skin, we found 740 upregulated (0.20–2.77) and 945 downregulated genes in IBH-LE (-0.20 - -2.14) (S1B Fig and S3 Table). Over 50% of DEGs were the same when IBH-LE was compared to IBH-NL or to H control skin: 29.4% of upregulated and 33.8% of downregulated genes were shared (Fig 2). In contrast, when we compared IBH-NL to H whole skin no differentially expressed genes were found (S1C Fig). For the above mentioned reasons the results presented from the study on whole skin were mainly derived from the comparison of IBH-LE to H control skin.

### Lesional whole skin of IBH-affected horses is characterized by changes in the epithelial barrier and substantial immune signatures

**Fig 3:**
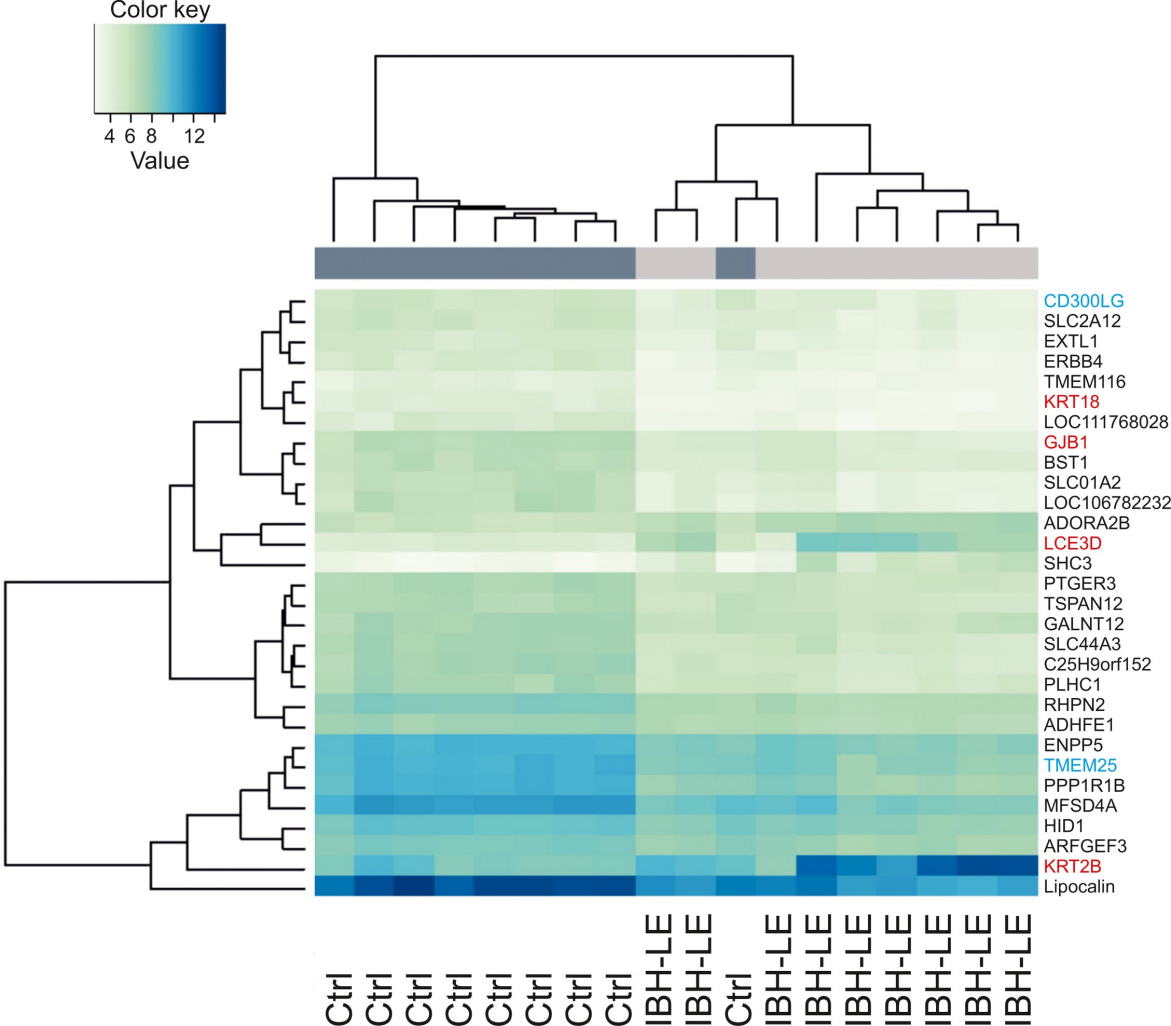
Hierarchical clustering top 30 DEGs expression of IBH-LE and H whole skin samples separates IBH-LE whole skin from H skin, with exception of one H control horse (lower mean counts are shown in light green and higher mean counts in dark blue). Epithelial barrier genes are listed in red and immune signature genes in blue.

Comparison of the IBH-LE and H skin showed a considerable transcriptional difference between the two studied groups (S1A Fig). Hierarchical clustering based on expression of the top 30 differentially expressed genes showed that IBH-LE and H samples clustered separately, with the exception of one healthy control horse. The majority of the top 30 genes were downregulated in IBH-LE. The log2 fold change of the top 30 genes ranged from -1.33 to -2.66 and from 1.67 to 2.90 with low p values (range 1.19 x 10^−17^ to 1.59 x 10^−13^). Among the top 30 DEGs, genes involved in terminal differentiation of keratinocytes, such as late cornified envelope protein 3D (*LCE3D*) as well as different types of keratins (e.g. *KRT2B*, *KRT18*) are noticeable (Fig 3), implying an involvement of these gene families in the pathogenesis of IBH. Other top upregulated genes were *LCE2B* and *KRT2B*, involved in epithelial barrier formation, *SHC3* and *ADORA2B* that has been shown to have a tissue protective role during hypoxic conditions. Other top downregulated genes were *LOC100056556*, the gene coding for the major allergen Equ c1, i.e. lipocalin, *PPP1R1B*, *C25H9orf152*, and *SLCO1A2*. However, these top downregulated genes did not belong to the enriched KEGG pathways nor gene ontology (GO) categories (Fig 3).

#### a) Changes in the epithelial barrier

**Fig 4:**
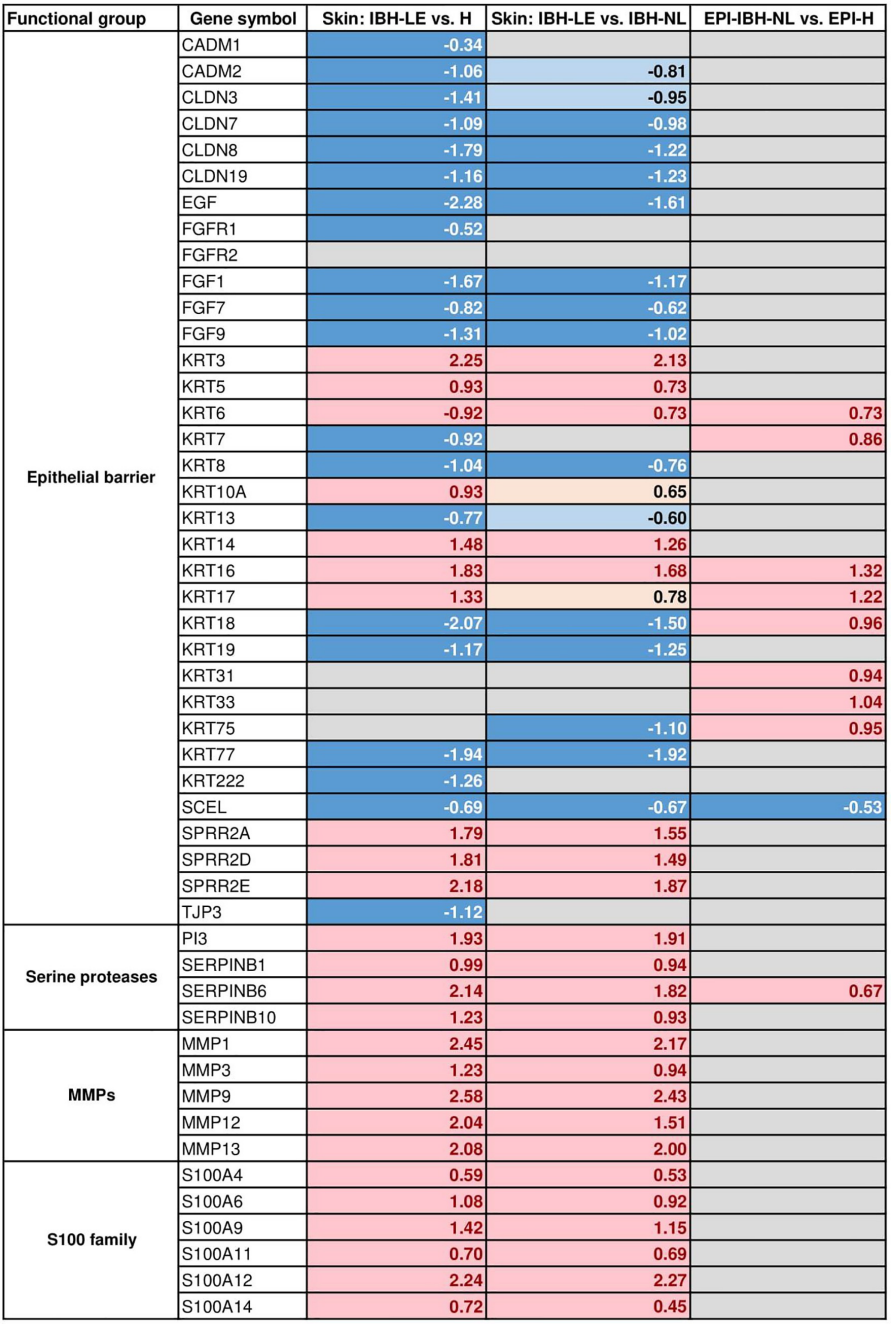
DEGs classified by gene families influencing epithelial barrier formation and maintenance in the three different transcriptome comparisons we studied: IBH-LE vs. H and IBH-LE vs. IBH-NL whole skin as well as non-lesional epidermis of IBH horses (EPI-IBH-NL) vs. epidermis from control horses (EPI-H). Only representative genes are shown. (Pink = statistically significant upregulation; beige = non-significant upregulation; dark blue = statistically significant downregulation; light blue = non-significant downregulation; gray = no difference in gene expression; false discovery rate <0.05). Log2 fold changes are noted for all DEGs.

GO analysis of DEGs between IBH-LE and H skin confirmed enrichment of genes affecting processes of epithelial cell differentiation, desmosome organization as well as regulation of epithelial morphogenesis, indicating important changes in barrier epithelium (Table 1 and S4 Table). Conversely to AD, most of the genes involved in terminal differentiation of keratinocytes, such as filaggrin, involucrin and loricrin showed high and comparable expression between IBH-LE and H (high mean counts; S2 Table). Small proline rich protein *SPRR2A*, another component of the cornified layer, as well as other members of SPRR family such as *SPRR2D* and *SPRR2E*, were significantly upregulated in IBH-LE. Furthermore, expression of certain keratins, such as *KRT3*, *-5*, *-6A*, *-6B*, *-10A*, *-6* and *KRT-17* was significantly upregulated, while the expression of *KRT8*, *-13*, *-15*, *-18*, *-19*, *-77* and *KRT-222* was significantly downregulated. In contrast, expression of genes involved in formation of adheres and tight junctions, such as *CADM1*, *CADM2*, *CLDN3*, *CLDN7*, *CLDN8* and *CLDN19* and *TJP3* were significantly downregulated (Fig 4).

**Table 1:** Selected biological processes enriched in IBH-LE whole skin compared to H whole skin, acquired by gene ontology analysis. Significantly enriched biological processes were ranked based on classic Fisher p values (p<0.05).

GO ID	Term	Annotated	Significant	Classic Fisher
GO:0030855	Epithelial cell differentiation	384	130	2.40E-05
GO:0048754	Branching morphogenesis of an epithelial . . .	118	40	0.01465
GO:0060688	Regulation of morphogenesis of a branchi . . .	42	16	0.03652
GO:0002934	Desmosome organization	8	6	0.00389
GO:0002467	Germinal center formation	11	6	0.03199
GO:0009620	Response to fungus	17	8	0.03699
GO:0035556	Intracellular signal transduction	1898	497	0.04769

Interestingly, expression of fibroblast growth factor receptor *FGFR1* as well as different fibroblast growth factors, namely *FGF7*, *FGF9* and *FGF1* was significantly downregulated in IBH-LE compared to H. *FGFR2* expression was, however, not different between IBH-LE and H skin. Additionally, expression of *EGF* was significantly downregulated (T). Many different serine proteases, such as *SERPINB1*, *SERPINB6* and *SERPINB11*, as well as matrix metalloproteinases like *MMP1*, *MMP3*, *MMP9*, *MMP12*, *MMP13* and others were significantly upregulated (Fig 4).

#### b) Immune cell signatures

**Fig 5:**
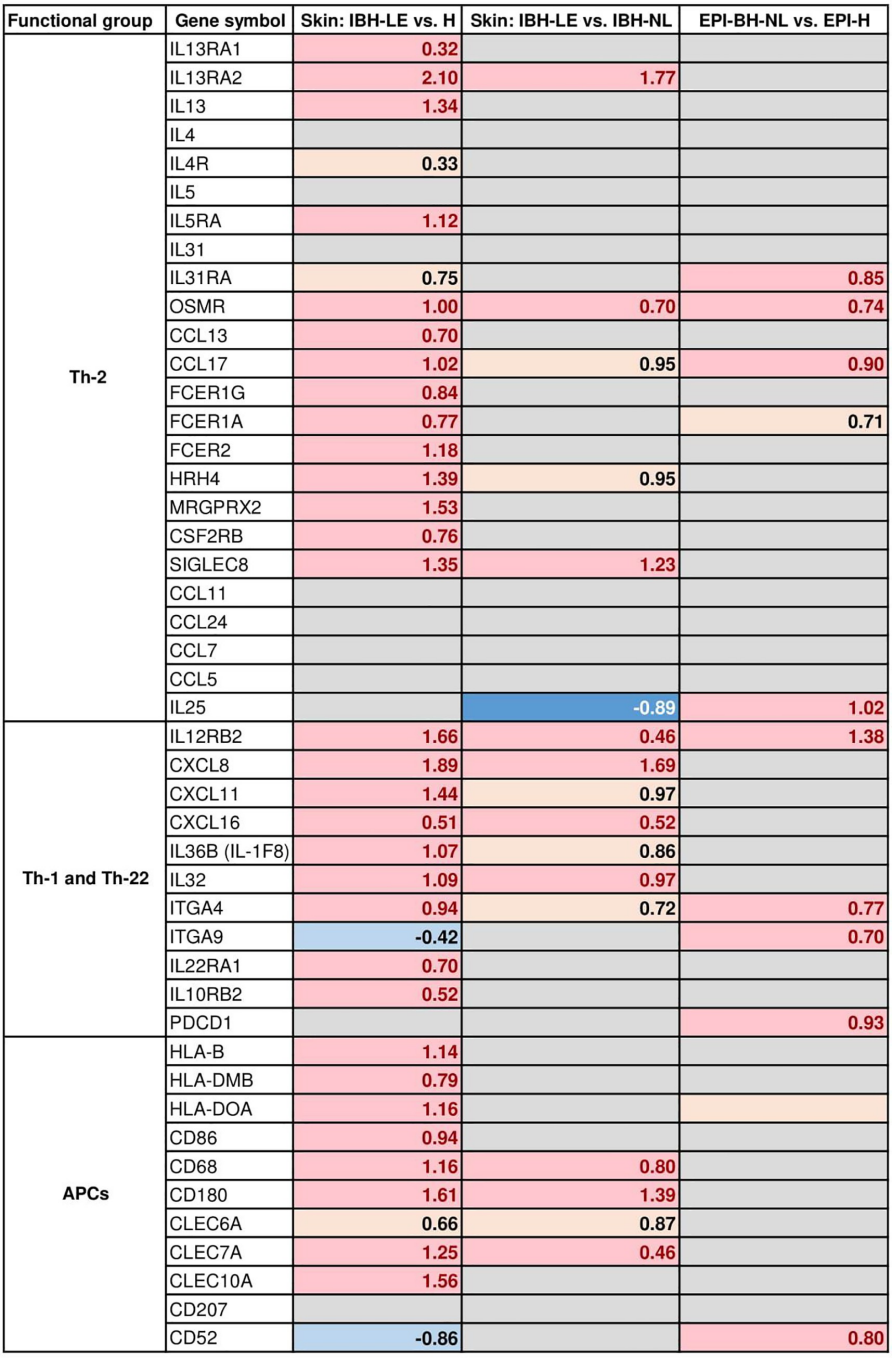
DEGs classified by gene families influencing immune signatures in the three different transcriptome comparisons we studied: IBH-LE vs. H and IBH-LE vs. IBH-NL whole skin as well as non-lesional epidermis of IBH horses (EPI-IBH-NL) vs. epidermis from control horses (EPI-H). Only representative genes are shown. (Pink = statistically significant upregulation; beige = non-significant upregulation; dark blue = statistically significant downregulation; light blue = non-significant downregulation; gray = no difference in gene expression; false discovery rate <0.05). Log2 fold changes are noted for all DEGs.

GO analysis also indicated involvement of categories related to different parts of the immune system, including response to fungus and formation of germinal center (Table 1 and S4 Table). Furthermore, gene set enrichment analysis (GSEA) using KEGG pathways indicated that genes of pathways FcεRI signaling (p = 0.01), Th-1 and Th-2 differentiation (p = 0.03), T and B cell receptor signaling (p = 0.02 and 0.007, respectively) and C-type lectin receptor signaling pathway (p = 0.003) are significantly overrepresented among DEGs (S5 Table). We therefore examined genes belonging to these respective pathways. How they possibly relate to cell populations involved in IBH is described below:

*Antigen presenting cells*. Two MHC class II genes homologous to *HLA-B* and *HLA-DMB*, and one MHC class I gene homologous to *HLA-DOA* were significantly upregulated. Additionally, expression of *CD86*, a T cell co-stimulator, as well as *CD68* and *CD180* were significantly upregulated. Furthermore, *CD209* (DC-SIGN), as well as certain c-type lectins, namely *C-LEC 6A*, *CLEC7A*, *CLEC10A*, were significantly upregulated in IBH-LE compared to H, suggesting increased numbers of antigen presenting cells, such as dendritic cells in IBH lesions. Moreover, expression of *FCER1G* and *FCER2* (CD23) which are suggestive of inflammatory dendritic epithelial cells (IDECs) was found to be significantly upregulated. Recently, it has been shown that IDECs also express histamine 4 receptor [22]. The gene coding for it, *HRH4*, was significantly upregulated in IBH-LE (Fig 5).

Expression of *FCER1A*, highly expressed on Langerhans (LC) and mast cells was also significantly upregulated. Expression of *CD207*, the gene encoding the langerin protein and marker of LCs, was not differentially expressed in IBH-LE compared to H. *FCER1G* and *FCER1A* form the FcεRI receptor and have previously been shown to be upregulated by Th2 cytokines, namely IL13. *FCER1G* and *MRGPRX2*, genes indicative of mast cells, were both significantly upregulated in IBH-LE compared to H skin (Fig 5).

*Th-2 cells*. The expression of *IL13*, a signature cytokine of Th-2 CD4+ T helper cells, as well as of both *IL13RA1* and *IL13RA2* was significantly upregulated in IBH-LE compared to H skin (Fig 5). Other Th-2 signature cytokines like IL-4 and IL-5 were not differentially expressed between the groups, and these cytokines were expressed on a very low level (S2 Table). However, both *IL5RA* (p = 0.01) and *IL4R* (p = 0.057) expression was upregulated. Furthermore, expression of Th-2 associated chemokines *CCL13* and *CCL17 was* significantly upregulated.

*Th-1 and Th-22 cells*. *IL12RB*, *CXCL8*, *CXCL11*, *CXCL16*, and *IL36B* (*IL-1F8*), indicative of Th-1 subsets, were also significantly upregulated. *IL22* was expressed on a very low level (S2 Table), and did not differ between IBH-LE and H skin. However, *IL22RA1* and *IL10RB2*, both subunits of IL-22 receptor were significantly upregulated in IBH-LE, as well as many genes of S100 family such as *S100A4*, *S100A6*, *S100A9*, *S100A11*, *S100A12*, and *S100A14* (Table 2). Expression of the cytokine *IL32*, recently described as a pluripotent inflammatory interleukin, was also significantly upregulated in IBH-LE skin [23] (Fig 5).

**Table 2:** Selected biological processes enriched in EPI-IBH-NL compared to EPI-H of control horses, acquired by gene ontology analysis. Significantly enriched biological processes were ranked based on classic Fisher p values (p<0.05).

GO ID	Term	Annotated	Significant	Classic Fisher
GO:0030216	Keratinocyte differentiation	74	15	1.10E-07
GO:0010718	Positive regulation of epithelial to mes . . .	32	7	0.00017
GO:0045616	Regulation of keratinocyte differentiati . . .	26	6	0.00038
GO:0007179	Transforming growth factor beta receptor . . .	114	13	0.00043
GO:1905331	Negative regulation of morphogenesis of . . .	11	4	0.00058
GO:1905332	Positive regulation of morphogenesis of . . .	21	5	0.00101
GO:0070328	Triglyceride homeostasis	15	4	0.06708
GO:0033153	T cell receptor V(D)J recombination	5	2	0.01369
GO:2001140	Positive regulation of phospholipid tran . . .	5	2	0.01369

*Eosinophils*. Since infiltration of eosinophils in the skin is one of the hallmarks of equine IBH, we investigated the expression of genes possibly involved in the infiltration of eosinophils. We found a significant upregulation of *siglec8*, as well as a significant upregulation of *CSF2RB*. *IL5RA*, which is expressed exclusively on eosinophils, was upregulated significantly in IBH-LE. However, expression of genes encoding for proteins involved in eosinophil infiltration, such as *CCL11*, *CCL24*, *CCL7*, *CCL5* or *CCR3* was not different between IBH-LE and H normal skin (Fig 5).

#### c) Response to hypoxia in lesional skin of IBH affected horses

**Fig 6:**
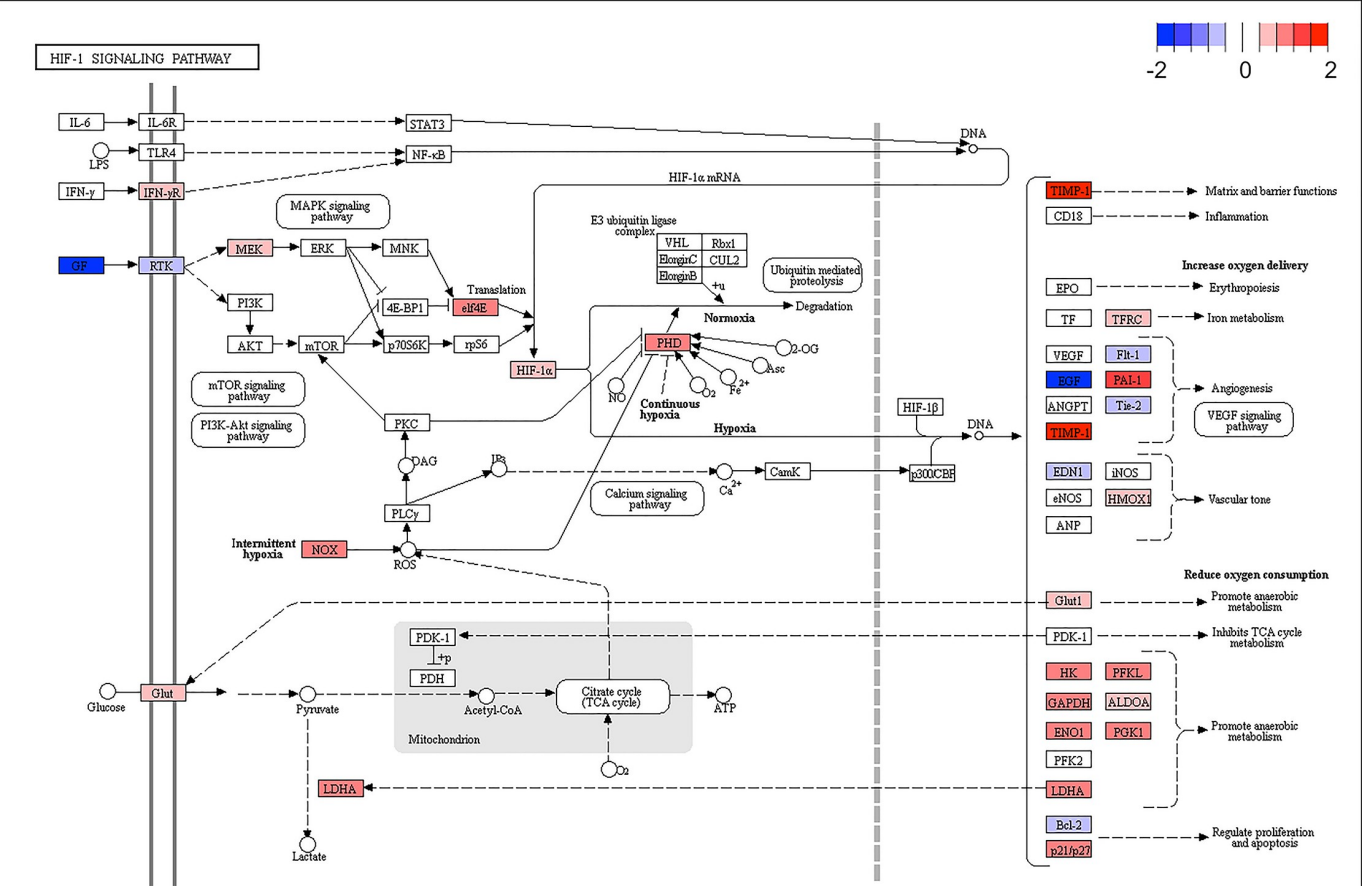
KEGG pathway "HIF-1-signaling pathway" in IBH-LE vs H skin comparison. Dark blue indicates downregulated genes (negative log2 fold changed) and red indicates upregulated genes (positive log2 fold changed).

**Fig 7:**
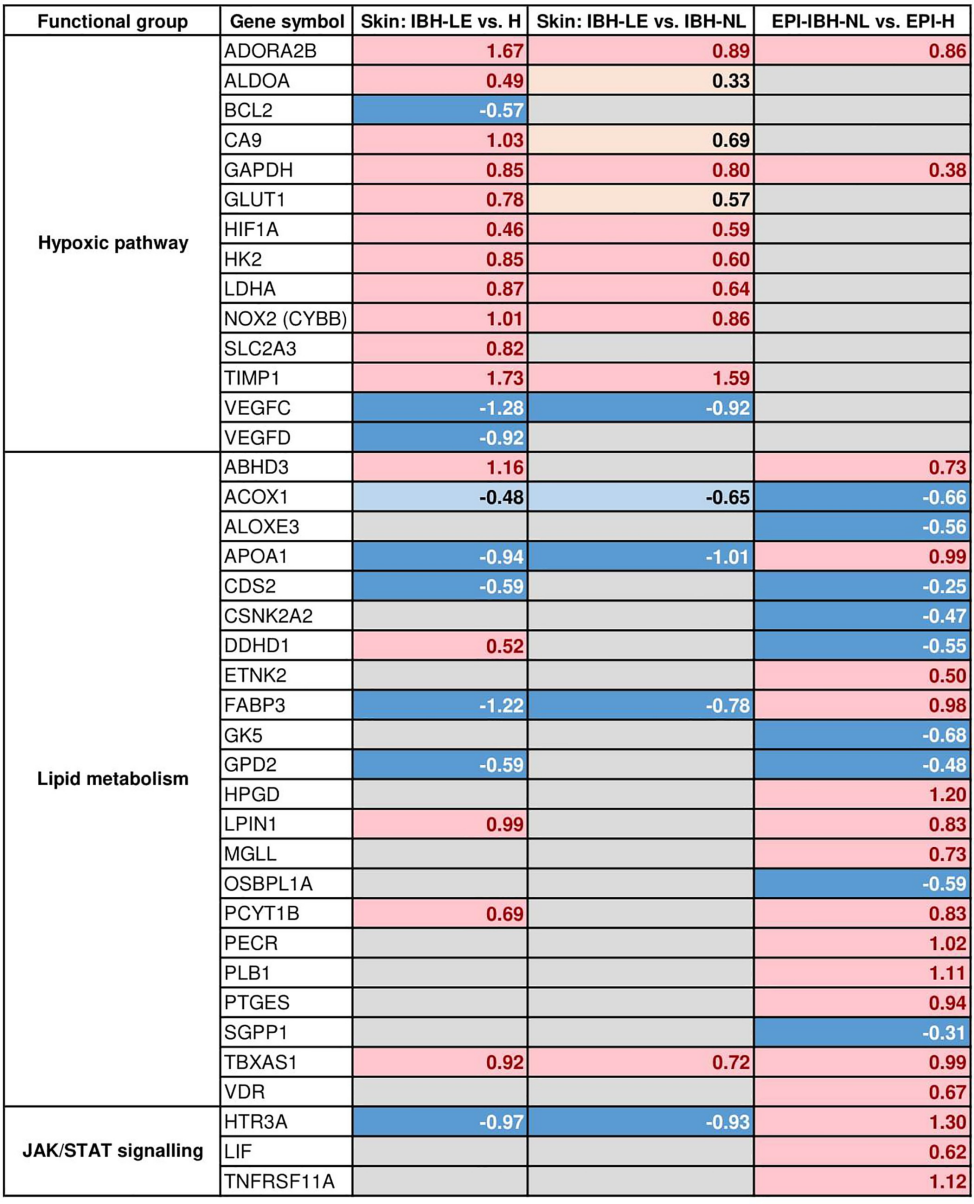
DEGs classified by gene families influencing HIF-1α mediated hypoxia and metabolism in the three different transcriptome comparisons we studied: IBH-LE vs. H and IBH-LE vs. IBH-NL whole skin as well as non-lesional epidermis of IBH horses (EPI-IBH-NL) vs. epidermis from control horses (EPI-H). Only representative genes from each family are shown. (Pink = statistically significant upregulation; beige = non-significant upregulation; dark blue = statistically significant downregulation; light blue = non-significant downregulation; gray = no difference in gene expression; false discovery rate <0.05). Log2 fold changes are noted for all DEGs.

GO and enrichment analyses revealed an enrichment of DEGs in the hypoxic pathway in IBH-LE compared to H skin (GSEA using KEGG pathways as gene sets; p = 0.005, S5 Table). We found a significant upregulation of *HIF1α*, a major sensor of cellular hypoxia, as well as of *NOX2 (CYBB)*, and of downstream effector molecules of HIF1α signaling, namely *SLC2A1 (GLU1)*, *SLC2A3 (GLUT3)*, *LDHA*, *CA9*, *HK2*, *TIMP1*, *GAPDH* and *ALDOA*, indicating an involvement of the hypoxic pathway in the pathogenesis of IBH (Fig 6). Interestingly, expression of *ADORA2B*, one of the top 30 DEGs, was also significantly upregulated (Figs 3 and 7).

### The transcriptome of non-lesional epidermis of IBH-affected horses differs from the epidermis of healthy horses

In order to investigate the baseline state of the epithelial barrier in IBH-affected horses, we studied the transcriptome of non-lesional epidermis from IBH-affected horses (EPI-IBH-NL) in comparison to epidermis from healthy controls (EPI-H).

Out of 33,078 annotated genes, 17,849 genes were found to be expressed in epidermis and were therefore used in the DEG analysis.

**Fig 8:**
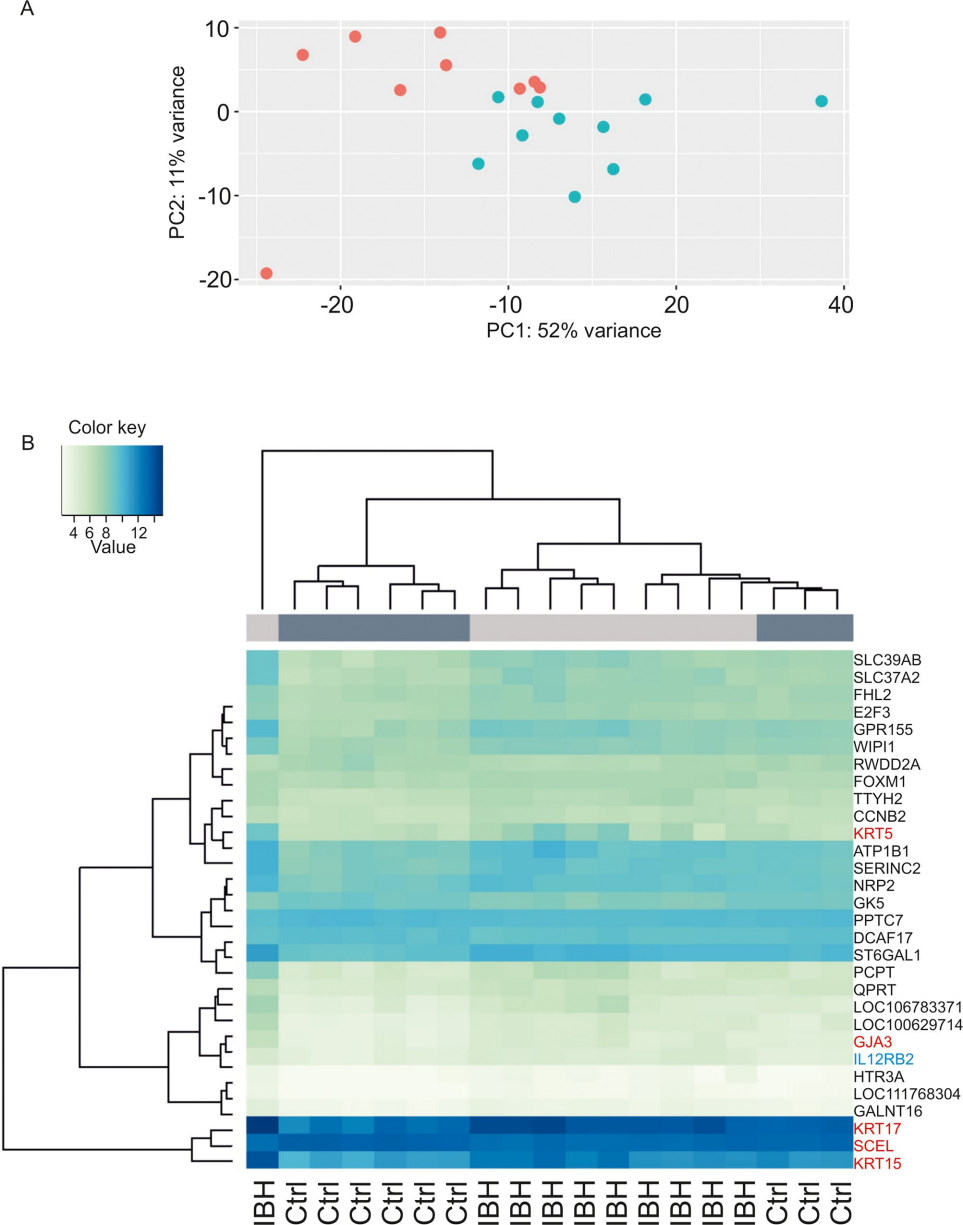
**(A)** Principal component analysis of top 500 most variable DEGs in EPI-IBH-NL (blue) and EPI-H (red) in the first two component spaces (p<0.05). **(B)** Hierarchical clustering of epidermis samples gene expression partially separates samples of EPI-IBH-NL from EPI-H. Three out of nine epidermis samples from control horses clustered with epidermal samples of IBH-horses (light green: lower mean counts, dark blue: higher mean counts). Epithelial barrier genes are listed in red and immune signature genes in blue.

PCA analysis of the epidermis samples derived from EPI-IBH-NL and EPI-H showed, despite considerable within-group variability, two distinctive clusters. However, three out of nine EPI-H clustered with the EPI-IBH-NL samples (Fig 8A). In EPI-IBH-NL 596 genes were differentially expressed. 461 genes were significantly upregulated (range log2 fold change 0.18–1.56) and 135 downregulated (-0.16 - -1.19) (S1D Fig and S6 Table).

#### a) Epithelial barrier

Hierarchical clustering of epidermis samples between EPI-IBH-NL and EPI-H was only partial, reflecting the results of PCA analysis. Among the top 30 DEGs in EPI-IBH-NL compared to EPI-H were genes involved in epithelial barrier formation and metabolism of epithelial lipids. Expression of *KRT5*, *KRT15*, *KRT17* and *GJA3* was upregulated while the expression of *GK5* and *SCEL* was downregulated (Fig 8B). Interestingly, although not among the top 30 DEGs, expression of other keratin genes was different between the two study groups. Expression of *KRT6B*, *KRT7*, *KRT16*, *KRT17*, *KRT31*, *KRT33* and *KRT75* was significantly upregulated (Fig 4). GO analysis results further confirmed enrichment of processes involved in keratinocyte differentiation and its regulation (Table 2 and S7 Table).

Besides enrichment of processes involved in keratinocyte differentiation and its regulation, GO analysis of DEGs showed enrichment of genes engaged in triglyceride homeostasis (Table 2 and S7 Table). This was particularly interesting, as terminally differentiated keratinocytes (corneocytes) and epidermal lipids function as "brick and mortar" in formation of the epithelial barrier of the skin. We next looked into KEGG pathway analysis, which confirmed that genes of glycerolipid metabolism were significantly overrepresented among DEGs in EPI-IBH-NL (p = 0.04) (S8 Table), suggesting changes in the epithelial barrier in IBH-affected horses already at this non-lesional stage.

#### b) Genes involved in pruritus

Interestingly, one of the genes among the 30 top DEGs in non-lesional epidermis of IBH horses is *HTR3A* (p = 0.003) coding for 5-hydroxytryptamine receptor 3A, shown to play a role in itch development [24] (Fig 8B). Furthermore, expression of both IL-31 receptor subunit genes, *IL31RA* and *OSMR* was significantly upregulated (Fig 5). IL-31 plays a major role in development of pruritus, and its signaling engages JAK/STAT pathway. Interestingly, in the non-lesional epidermis of IBH horses, two additional genes, *LIF* and *TNFRSF11A* (RANK), engaging JAK/STAT pathway were also significantly upregulated (Fig 7).

#### c) Immune signatures

Unlike in IBH-LE skin, non-lesional epidermis of IBH horses is not characterized by a strong immune cell signature. Nonetheless, we found a significantly upregulated expression of *CCL17 (TARC)*, *ITGA4*, *ITGA9*, all involved in T cell homing (Fig 5). Furthermore, *IL12RB2*, expressed on T cells, was among the highest upregulated genes in EPI-IBH-NL compared to EPI-H (Fig 8B). *CD52*, expressed on mature lymphocytes as well as monocyte derived dendritic cells, was significantly upregulated as well. However, there was no evidence of Th-2 responses in EPI-IBH-NL, with the notable exception of *IL25*, which was significantly upregulated (Fig 5). Additionally, KEGG pathway analysis indicated that genes of cell adhesion molecules (p = 0.009), cytokine-cytokine receptor interaction (p = 0.009) and antigen processing and presentation pathways (p = 0.04) were significantly overrepresented in non-lesional epidermis of IBH-affected horses (S8 Table).

## Discussion

In the present study, we aimed to characterize the reaction and state of the epithelial barrier in horses suffering from IBH, as well as immune responses possibly involved in the pathogenesis of this disease. Therefore, we first studied the transcriptome of IBH-LE and IBH-NL whole skin in comparison to H controls. We found a high number of DEGs which indicated that IBH-LE is characterized by changes in the epithelial barrier, substantial changes immune signature and a strong involvement of the hypoxic pathway.

Our data showed a significant downregulation in the group of genes involved in the formation of tight junctions in the skin. Differing from human AD patients, genes coding for proteins involved in terminal differentiation of keratinocytes (*FLG*, *IVL*) were not differentially expressed between our study groups (S2 Table). Taken together, our data suggests that lesional skin of horses with IBH is characterized by alterations in adherens junctions, and not by deficiency in terminal differentiation in keratinocytes, as in human AD [8]. Yang *et al*. have described a fibroblast growth factor receptor 1 and 2 (*fgfr1*, *fgfr2*) knock out mouse model that develops skin lesions similar to those in patients with AD, particularly with regard to the inflammatory infiltrate and the epidermal thickening. In their work, they elaborately showed how FGFs regulate tight junction components and how keratinocyte hyperproliferation is most likely mediated by IL-1F8 (CD36B) and S100A9 [25]. Similarly to this mouse model, lesional IBH-skin is characterized by epidermal thickening, and, as our data suggests, tight junction disruption. In IBH-LE skin expression of *FGFR1* was significantly downregulated, while the expression of *FGFR2*, which is in mice the most important receptor in lesion development, was not affected. However, in IBH-LE we found expression of fibroblast growth factor 7 (*FGF7*), encoding one of the high affinity ligands to FGFR2, as well as *FGF9* and *FGF1* to be significantly downregulated, thus impairing FGFR2 signaling Additionally, genes encoding both IL-1f8 and A100A9 were upregulated in IBH-LE skin, implying that similar mechanisms might be involved in epidermal thickening and disruption of epithelial barrier in horses with IBH. However, the exact mechanism remains to be elucidated.

Multiple serine protease as well as matrix metalloproteinase transcripts were upregulated in IBH-LE, possibly leading to further destruction of the skin integrity. Interestingly, similarly to human AD, *MMP9* was one of the upregulated matrix metalloproteinase genes. In AD patients overexpression of *MMP9*, *MMP10* and *S100A7A* was observed, and the link between MMP function and disruption of epithelial barrier has been shown [9, 26–29]. In particular, MMP9 seems to play a role in the development of epidermal inflammation. Furthermore, it has been shown that the activity of MMP9 is induced by IL-13 [30]. Interestingly, *IL13* was the only Th-2 cytokine found to be upregulated in IBH-LE compared to healthy skin. These findings indicate similar mechanisms in equine IBH and human AD, where IL-13 has been suggested to be the key Th-2 cytokine driving inflammation in the periphery while the effect of IL-4 is more central, as reviewed in Bieber 2019 [31–34].

Our data indicates that both Th-1 and Th-2 responses seem to be involved in IBH-LE (Fig 5). This is similar to human AD skin lesions, where both Th-2 and Th-1 signatures as well as strong inflammatory mediators were found [9]. Furthermore, a prominent feature of IBH-LE skin was inflammatory dendritic epithelial cells (IDEC) signature. We found upregulation of *CD209* (DC-SIGN), *FCER1*, and *CD11C* (p<0.03); these markers strongly differentiate IDEC from plasmacytoid DCs. Additionally, we found upregulation of *HRH4*, encoding the histamine 4 receptor, another receptor correlated with IDECs [22]. On the other hand, genes encoding markers of LCs such as CD207 (Langerin) or CD83 were not differentially expressed between IBH-LE and H skin, although previous studies using electron microscopy of skin lesions had indicated an increase of LCs in IBH [5, 35, 36].

Upregulation of *SIGLEC8*, *CSF2RB* and *IL5RA* in IBH-LE skin is in alignment with the presence of eosinophils in our samples, as demonstrated by the histopathological evaluation. However, our study did not allow to elucidate which mechanisms might be responsible for their influx into the skin, as none of the eotaxins were differentially expressed between IBH-LE and H skin. Recently, Nobs *et al*. have shown that in the setting of allergic airway inflammation, together with IL5, GM-CSF intrinsically controls eosinophil accumulation [37]. Interestingly, in IBH-LE, *CSF2RA*, the gene coding for the GM-CSFRα subunit and *CSF2RB*, coding for the GM-CSFRβ subunit, were significantly upregulated. The two GM-CSF transcripts, however, were expressed at a very low level and the expression did not differ between the study groups. Whether this pathway is involved in eosinophil infiltration in IBH-LE should be further investigated.

KEGG and Reactome pathway analysis revealed an enrichment of DEGs in the hypoxic pathway in IBH-LE. In particular *HIF1A* expression was significantly upregulated in IBH-LE (Figs 6 and 7). HIF-1α is a major cellular sensor of low oxygen levels (hypoxia), the expression of which can be induced by hypoxia itself as well as by environmental conditions associated with pathological stress such as inflammation, bacterial infection or cancer [38]. In a pathologically hypoxic immune environment, such as IBH-LE skin, HIFs can play a crucial role in modulating immune cell effector functions [39–41], increasing survival and functionality of eosinophils, mast cells and basophils, the main effector cells of type I hypersensitivities. Moreover, HIF-1α has a major impact on DCs by increasing their survival and functions [42–47]. Additionally, HIFs can induce late stage maturation of ILC2s, which significantly contribute to allergic inflammation [48, 49]. Taken together, our data suggest that the upregulation of *HIF1A* and its impact on downstream targets such as *GLUT1*, *ALDO*, *LDHA* and *CA9* strongly influences and possibly further promotes allergic inflammation. Additionally, it was shown that HIF-1α is the transcriptional regulator of ADORA2B, the adenosine A2b receptor. ADORA2B plays a central role in tissue adaptation to hypoxia and its transcript was found to be among the top five most significantly upregulated genes in IBH-LE (Figs 3 and 7) [50, 51]. Moreover, pharmacologic inhibition of HIFs has proven to ameliorate allergic contact dermatitis in human patients [52].This approach might thus also be a treatment option for IBH, but the exact effects of HIF-1α on effector cells in IBH needs first to be investigated. To our knowledge, this is the first indication of HIF-1α-mediated hypoxia pathway involvement in IgE-mediated skin allergy.

While a high number of genes were differentially expressed in IBH-LE compared to H whole skin, no significant DEGs were found when comparing IBH-NL whole skin to H control skin. In contrast, comparison of only the epidermis (EPI-IBH-NL vs EPI-H) revealed 461 significantly upregulated and 135 significantly downregulated genes. These genes were partly the same genes as those identified when comparing IBH-LE skin to IBH-NL or H control skin (Figs 4, 5 and 7). This finding suggests that there are indeed DEGs between IBH-NL and H skin, which, however, could not be detected when transcriptomes of whole skin were compared. The most probable causes of this discrepancy are the larger number of genes which are expressed in the whole skin compared to epidermis only (21,337 and 17,849 genes, respectively) and the more complex cellular composition. The whole skin of horses consists of a relatively thick dermis, comprising different cells and structures such as fibroblasts, adnexal structures, blood vessels and nerves of which some are probably not involved in the IBH pathogenesis. Due the smaller proportion of the relevant cells, weaker differences in gene expression have to be expected when comparing NL-IBH skin to H control skin and may thus not reach statistical significance.

In order to study the "baseline state" of epithelial barrier in IBH-affected horses, we compared the transcriptomes of EPI-IBH-NL to EPI-H. Unlike in IBH-LE, hierarchical clustering of epidermal transcriptomes (Fig 8B) was only partial, reflecting the results of PCA analysis. This is probably due to the fact that the differences in the transcriptomes of NL and H epidermis were substantially smaller than when comparing IBH-LE to H skin and thus inter-individual differences had a stronger effect. Larger numbers of samples and a better phenotyping of the individuals included in the study might thus have been needed. Finally, the three control horses that clustered with the IBH-affected group might have been predisposed for IBH and might even develop IBH later in life. Two out of three horses clustering with IBH-affected horses were very young (6 and 24 months old, respectively) leading to low reliability of their status as controls.

Nevertheless, comparison of the transcriptome between EPI-IBH-NL and EPI-H resulted in valuable findings for our understanding of the pathogenesis of IBH. GO analysis revealed that keratinocyte differentiation and lipid homeostasis are enriched with DEGs. KEGG pathway analysis further confirmed that genes of the glycerolipid metabolism pathway are significantly enriched among DEGs (p = 0.04, S8 Table). A functional epidermal skin barrier requires the formation of a cornified envelope from terminally differentiated keratinocytes and lipids [53]. The most abundant lipid families in the *stratum corneum* are ceramides, cholesterol and fatty acids, and disruption in their content may cause permeability defects of the barrier [54]. Expression of *SGPP1*, encoding an enzyme involved in sphingolipid *de novo* synthesis was significantly downregulated, suggesting an impairment in ceramide production. It has been shown in human AD that decreased epidermal activity of sphingomyelinase (A- and N-SMase), also involved in ceramide synthesis, is decreased in lesional and nonlesional skin, correlating with a disturbed barrier function. Moreover, expression of *GK5* and *GPD2* that play a role in glycerolipid metabolism was also significantly downregulated in our study. Interestingly, downregulation of *GPD1*, a member of the GAPDH family, in lesional skin of AD patients has been shown in multiple studies [55].

Most interestingly, analysis of the transcriptome of EPI-IBH-NL suggests a propensity for itch development. Even in clinically unaffected skin sites, expression of both genes encoding the IL-31 receptor subunits, *IL-31RA* and *OSMR*, as well as of *HTR3A*, shown to play a role in pruritus development were upregulated [24]. Involvement of IL-31 in IBH pathogenesis has been supported by recent finding of Olomski *et al*., showing that targeting IL-31 significantly ameliorates clinical signs of IBH [56]. The authors also found upregulation of IL-31 mRNA in IBH-LE which we have not observed in our study. Expression of all Th-2 cytokines, except *IL13*, was very low in our study (S2 Table). Taken together, our data suggests an impairment in epidermal lipid synthesis, which can in turn lead to a disruption of the epithelial barrier and, combined with a propensity for itch development, possibly predisposes horses for IBH. Furthermore, *IL25* upregulation could suggest potential activation of ILC2s that subsequently contribute to type I hypersensitivity development. Additionally, the gene encoding TNF receptor super family member 11a, *TNFRSF11A*, was significantly upregulated in EPI-IBH-NL. This finding is particularly interesting as Velie *et al*. identified the *TNFRSF11A* gene as potential genetic risk factor for IBH development [57].

In conclusion, our study has highlighted common features in human AD and equine IBH, confirming the value of this disease as a natural model of AD.

New mechanisms identified in our study, such as immune-mediated alterations in the epithelial barrier and *IL13* upregulation in IBH-LE, as well as lipid metabolism impairment in EPI-IBHNL are most probably important in the pathogenesis of IBH and could potentially be explored as new therapeutic targets, once their relevance is confirmed.

## Supporting information

S1 Fig (TIF)

S1 Table (XLSX)

S2 Table (XLSX)

S3 Table (XLSX)

S4 Table (XLSX)

S5 Table (XLSX)

S6 Table (XLSX)

S7 Table (XLSX)

S8 Table (XLSX)
